# Subtropical and flower crops breeding at the Subtropical Scientific Centre

**DOI:** 10.18699/VJ21.047

**Published:** 2021-07

**Authors:** A.V. Ryndin, R.V. Kulyan, N.A. Slepchenko

**Affiliations:** Federal Research Centre the Subtropical Scientific Centre of the Russian Academy of Sciences, Sochi, Russia; Federal Research Centre the Subtropical Scientific Centre of the Russian Academy of Sciences, Sochi, Russia; Federal Research Centre the Subtropical Scientific Centre of the Russian Academy of Sciences, Sochi, Russia

**Keywords:** biodiversity, genetic collection, subtropical and flower crops, breeding, биоразнообразие, генетическая коллекция, субтропические и цветочные культуры, селекция

## Abstract

This paper presents the results on the breeding work carried out by the Subtropical Scientific Centre of the Russian Academy of Sciences. Currently, the Centre’s breeders are doing a lot of work aimed at breeding new fine yielding cultivars of subtropical and flower crops that will be resistant to growing conditions; they include kaki persimmon, feijoa, mandarin, freesia, crown anemone, pelargonium and chrysanthemum. The sources of high-level priority traits in flower crops that are valuable for further breeding in order to improve decorative (colour, flower shape, inflorescence), economic and biological traits (flowering period, a large number of flowers in the inflorescence, storage period of cut flowers, disease resistance, high reproduction coefficient) were recorded. The aim of the research is to improve the subtropical and flower crops assortment. The objects of the research were 989 hybrid forms: 136 citrus crops, 56 persimmon, 36 feijoa, 38 tea plant, 11 pear, 24 hazel, 108 freesia, 398 crown anemone, 120 pelargonium and 62 chrysanthemum hybrids. New cultivars with a complex of valuable traits have been created as a result of the scientific work. Over the past five years, FRC SSC of RAS has created 50 new cultivars: 26 pelargonium, 15 anemone, 5 freesia, 2 chrysanthemum, 1 persimmon and 1 apple and submitted them to the State Cultivar Commission. The “State Register of Selection Achievements Authorized for Use for Production Purposes” has included 63 cultivars developed by FRC SSC RAS, including 26 pelargonium, 13 anemone, 9 chrysanthemum, 7 freesia, 4 hazel, 3 feijoa and 1 tea plant cultivars. 46 patents for breeding achievements have been obtained.

## Abstract (ru)

Представлены результаты селекционной работы ФИЦ «Субтропический научный центр Российской академии наук» (ФИЦ СНЦ РАН). Селекционерами ФИЦ СНЦ РАН проводится большая работа, направленная на выведение новых урожайных, высококачественных, устойчивых к биотическим и абиотическим факторам сортов субтропических и цветочных культур: хурмы восточной, фейхоа, мандарина, фрезии, анемоны корончатой, пеларгонии и хризантемы. Выделены источники хозяйственно ценных признаков цветочных культур, которые представляют интерес для селекции на улучшение декоративных (окраска, форма цветка, соцветия) и хозяйственно-биологических признаков (период цветения, большое количество цветков в соцветии, период стояния в срезе, устойчивость к болезням, высокий коэффициент размножения). Цель исследований – совершенствование сортимента субтропических и цветочных культур. Объектами исследований служили 989 гибридных форм, из них цитрусовых – 136, хурмы – 56, фейхоа – 36, чая – 38, груши – 11, лещины – 24, фрезии – 108, анемоны корончатой – 398, пеларгонии – 120, хризантемы – 62. Результатом работы стало выведение сортов с комплексом ценных признаков. За последние пять лет в ФИЦ СНЦ РАН создано и передано в Госсорткомиссию РФ 50 новых сортов, в том числе 26 – пеларгонии, 15 – анемоны, 5 – фрезии, 2 – хризантемы, 1 – хурмы и 1 – яблони. В «Государственный реестр селекционных достижений…» включено 63 сорта селекции ФИЦ СНЦ РАН, в том числе 26 сортов пеларгонии, 13 – анемоны, 9 – хризантемы, 7 – фрезии, 4 – фундука, 3 – фейхоа, 1 – чайного растения. Получено 46 патентов на селекционные достижения.

## Introduction

The humid subtropical zone on the Black Sea coast of Krasnodar Territory is the only suitable zone in Russia for cultivating subtropical fruit crops, citrus crops and tea plant (Ryndin, Tereshkin, 2012; Tutberidze, 2015; Ryndin, 2016). One of the main objectives for the development of subtropical fruit growing in this zone is to expand and improve the subtropical fruit and flower crops assortment. It should be noted that interest in these crops is also increasing in other regions of the Russian Federation, thanks to the development of greenhouse, indoor and office gardening, as well as the ongoing work carried out to create geographical areas and expand the subtropical crops range (Ryndin, Tuov, 2010; Pchikhachev, Korzun, 2017; Collections..., 2019; Omarov et al., 2020).

Currently, the flower crops cultivation in our country is mainly based on imported assortment. The Black Sea coast is promising for cultivating cut flowers in the winter-early spring period, when the overall flower assortment is small and the population’s demand for these products is greatly increasing. One of the strategy principles in developing flower and ornamental crops production, including import substitution, is to apply the latest breeding achievements (Ryndin et al., 2015). The creation of new highly decorative forms that will be productive, competitive, environmentally hardy, very early and late-flowering, original in flower shape, and rare in colour, remains an urgent task for flower crop breeding (Gutiyeva, 2014, 2015; Mokhno et al., 2014; Bratukhina, Paschenko, 2015; Kozina, 2015, 2018).

The climatic conditions in the given zone allow us to create collections and carry out the breeding work, both in open ground conditions and in glass greenhouses without additional heating, in many flower crops (tulip, chrysanthemum, hippeastrum, pelargonium, freesia, crown anemone, and others). The main methods of creating material for breeding were and still are intervarietal and interspecific crosses, clonal selection and selection on a nucellar basis. New cultivars are more flexible and resistant to adverse environmental conditions. The essential significance of the local cultivars is provided by their high adaptive potential.

Federal Research Centre the Subtropical Scientific Centre of the Russian Academy of Sciences – FRC SSC of RAS (earlier Russian Research Institute of Floriculture and Subtropical Crops) is one of the oldest scientific institutions of our country: in 2019 it celebrated its 125th anniversary. From the first days of the institution’s existence, the research program included the following issues that are still relevant today: creation and maintenance of subtropical, fruit and flower crops collections, introduction and study of the possibility to cultivate new species and cultivars for the zone. The rich genetic collections of both cultivated plant species and their wild relatives are collected on the basis of FRC SSC of RAS, which helps to preserve economically valuable species and forms. They are the objects of diverse research that contributes to the in-depth study of ecological and biological features, the development of new technological cultivation methods, the solution of plant protection issues and the allocation of material for further breeding work (Omarov et al., 2014, 2020; Ryndin, Kulyan, 2016; Kulyan et al., 2017; Volk et al., 2018; Collections…, 2019; Ryndin, Slepchenko, 2019).

**Fig. 1.**
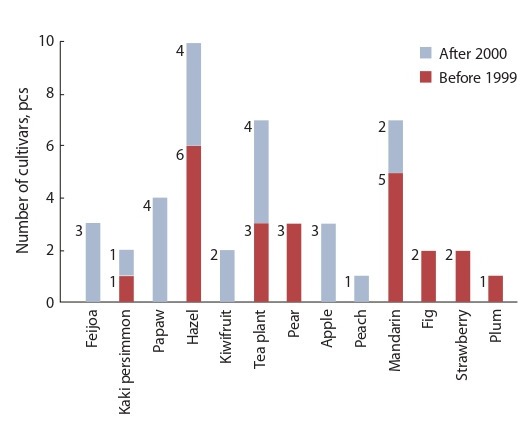
The number of subtropical, southern fruit, berry and tea cultivars, created in FRC SSC of RAS.

**Fig. 2.**
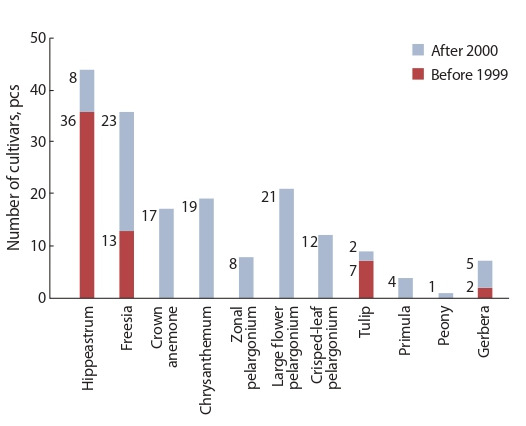
The number of flower cultivars, created in FRC SSC of RAS.

The breeding work was begun in the FRC SSC of RAS in 1930. The number of subtropical, southern fruit, berry and tea cultivars, which have been bred and recommended for production and use, is shown in Fig. 1, and flower cultivars – in Fig. 2. Some of them are used in the production and landscaping not only within the region, but also in other parts of southern Russia.

Currently, the work to create new cultivars of mandarin, kaki persimmon, feijoa, pear, tea plant, freesia, crown anemone, pelargonium and chrysanthemum continues (Loshkareva, 2014; Kulyan, Omarova, 2018; Kiseleva, 2020; Omarov, Omarova, 2020; Yakushina, 2020). 

The purpose of this paper is to analyze the main collection samples and the development of new cultivars that meet the requirements of intensive gardening, and to identify promising fields in breeding, allowing us to improve the assortment of humid subtropics in Russia.

## Materials and methods

**Fig. 3.**
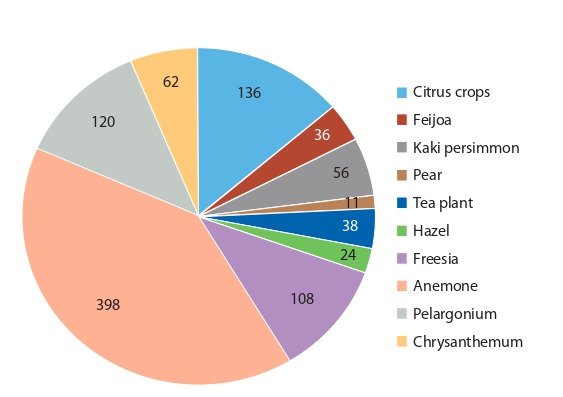
Crop hybrid fund of FRC SSC of RAS, pcs.

For this purpose, the hybrid fund of subtropical, southern fruit and flower crops is currently being studied at FRC SSC of RAS (Fig. 3). The objects of research are: hybrid forms of citrus crops (frost resistance, resistance to biotic stressors, productivity) presented in the amount of 136, 50 of which are promising, 24 are elite; kaki persimmon (resistance to biotic and abiotic factors) – 56, 18 of which are promising; feijoa (high yield, high fruit quality, early ripening) – 36, 7 of which are elite; pear (high productive and adaptive potentials) – 11, 2 of which are promising; tea plant (productivity, high biochemical (tannin not less than 26 %) and organoleptic indicators) – 38 forms, 3 of which are elite and 5 are winter-hardy; hazel – 24 promising forms; flower crops (decorativeness, productivity, abundant flowering): freesia – 108, 6 of which are promising; anemone – 398, 98 of which are promising; pelargonium – 120, 80 of which are promising, 16 are elite; chrysanthemum – 62, 10 of which are promising, 6 are elite.

New subtropical fruit cultivars should have the following parameters: low- and medium-grown, a high rate of productivity and adaptation to growing conditions, resistance to pests and fungal diseases, a stable yield, and fruits of high commercial and taste qualities (Omarov et al., 2018). Concerning flower crops, they should have decorativeness, abundant long-term flowering, high productivity and reproduction rate, as well as resistance to specific environmental conditions (Gutiyeva, 2020; Paschenko, 2020a, b). 

In the process of research were used: the methods of State cultivar testing for agricultural and ornamental crops; programs and methods for studying fruit, berry and nut cultivars: “Program of the North-Caucasian Centre for the Breeding of Fruit, Small-fruit, Ornamental Crops and Grapevine for the Period until 2030” (2013); “Modern Methods and Tools for the Assessment and Selection of Breeding Material of Orchard Crops and Grapevine” (2017). Parent pairs were selected according to “Citrus Fruit Breeding. VIR Guidelines” (1989). The primary and competitive study of crown anemone hybrids was carried out according to the “Protocol for Tests for the Distinguishability, Uniformity, and Cultivation Stability of the Poppy Anemone” (2003); for chrysanthemum, crosses were carried out according to I.A. Zabelin’s method (1975).

## Results and discussion

Citrus crops (Citrus family Rutaceae)

Breeding program for the creation of winter-hardy cultivars is based on remote hybridization, the main donors are Citrus trifoliata L. (syn. Poncirus trifoliata (L.) Raf.), Citrus japonica Thunb. (syn. Fortunella margarita (Lour.) Swingle), Citrus cavaleriei H. Lév. ex Cavalerie (syn. Citrus ichangensis Swingle), C.×insitorum Mabb., as well as previously obtained interspecific hybrids. Medium-grown, winter-hardy, semi-deciduous genotypes, not exceeding 2.0 m in height at the age of ten, were identified.

The program for breeding cultivars with high fruit quality (large-fruited, leveled, with high sugar-acid index, seedless, different in ripening terms) is a leading direction in breeding and is based on applying interspecific crosses (C. reticulata× C. sinensis; C. reticulata×C. paradise; C. reticulata×C. maxima). Genotypes with different expressed levels of traits were obtained on the basis of parental forms of different geographical origin, which creates prerequisites for expanding the genetic basis while creating cultivars with high ecological adaptiveness and a complex of other economically valuable traits.

At the moment, the study includes 136 forms of citrus crops, 50 of which are promising and 24 are elite. Forms with high fruit quality occupy the leading place in the hybrid fund collection and represent the main material for breeding (Table 1).

**Table 1.**
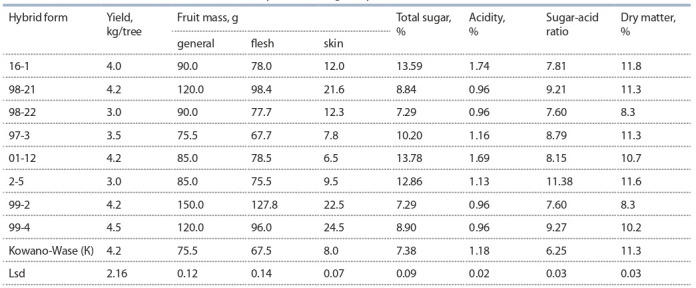
Characteristics of the selected mandarin hybrid forms aged 5 years Notе. C – control.

Sugar and acidity levels are the main criteria for the overall fruit quality. Sugar-acid ratio characterizes the degree of fruit sweetness, i. e. a harmonious taste of mandarin fruits is achieved with a certain sugar-acid ratio. The fruits of hybrids 2-5, 99-4, 98-21 and 97-3 have the best taste qualities compared to the control Kowano-Wase (see Table 1).

In many citrus-growing countries, where productivity, early ripening, fruit quality, and immunity to viral diseases are foremost priorities, breeding is carried out on the basis of nucellar polyembryonia (Nesumi et al., 2001; Ben-Hayyim, Moore, 2007; Ali et al., 2013; Combrink et al., 2013; Yasuda et al., 2015).

The FRC SSC of RAS also conducts a breeding program aimed at obtaining high-yielding, early-maturing, mediumgrown and variegated forms using nucellar seedlings. 12 promising forms were recorded (Table 2).

**Table 2.**
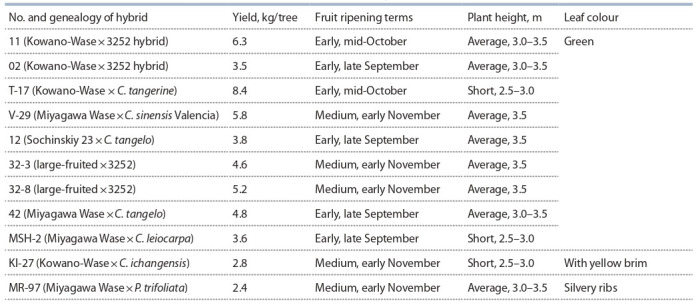
Characteristics of promising nucellar mandarin seedlings aged 5 years

**Fig. 4.**
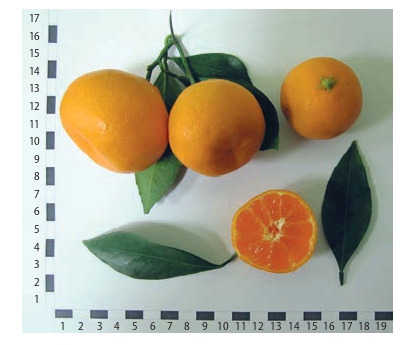
Seedling 02 (C. reticulatа × hybrid 3252).

**Fig. 5.**
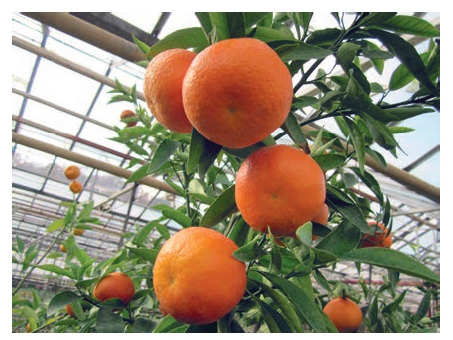
Seedling Т-17 (C. reticulatа × C. tangerine).

Among the nucellar seedlings, the most valuable are lowand medium-grown early-ripening hybrids, which have good fruit quality (Fig. 4, 5).

**Fig. 6.**
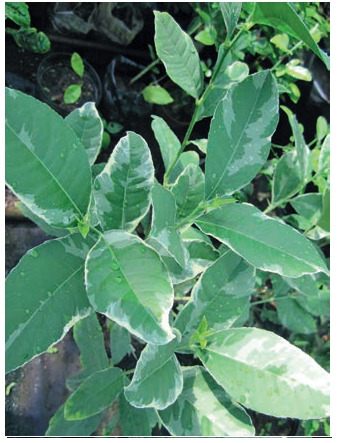
Seedling MR-97 (C. reticulatа×P. trifoliatа)

**Fig. 7.**
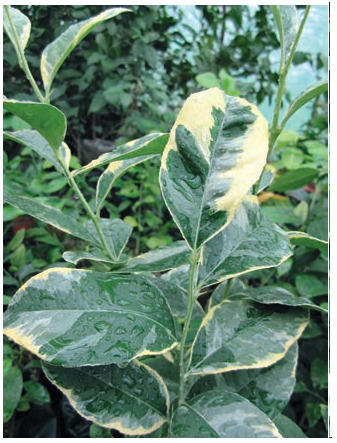
Seedling КI-27 (C. reticulatа× C. ichangensis).

Variegated forms are becoming very popular among citrus lovers and collectors. We have identified nucellar seedlings MR-97 (Fig. 6) and KI-27 (Fig. 7), which do not lose this trait during vegetative reproduction.

**Fig. 8.**
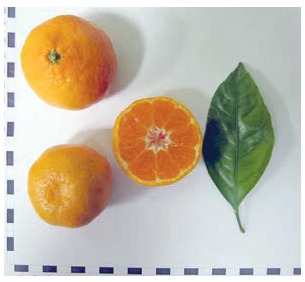
Mandarin Clone 22.

**Fig. 9.**
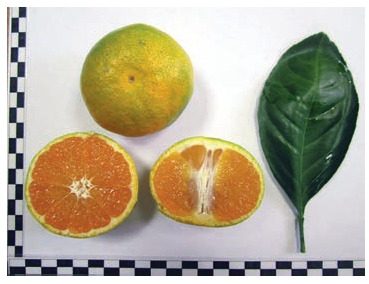
Mandarin Clone 33.

A number of bud mutations were recorded on the basis of the mandarin genetic collection of FRC SSC of RAS, the altered traits were fixed in the process of reproduction by budding, and two isolated clones were subjected to State cultivar testing (Fig. 8, 9).

Kaki persimmon (Diospyros kaki L.) is considered one of the most frost-resistant subtropical crops. Without significant damage, adult plants can withstand prolonged low temperatures up to –12…–15 °С. The main goal of kaki persimmon breeding is to create high yielding cultivars with fine fruit quality, (fruit weight 150–200 g, sugar amount 15–20) and resistance to extreme environmental factors. Over the past 10 years, 16 intervarietal and 5 interspecific cross combinations have been carried out, the best ones have been determined: Djiro×Geili, Djiro×Zenji-Maru and Hiakume× Fuyu, Djiro×D. virginiana L., of which the largest number of promising hybrids was identified (Table 3).

**Table 3.**
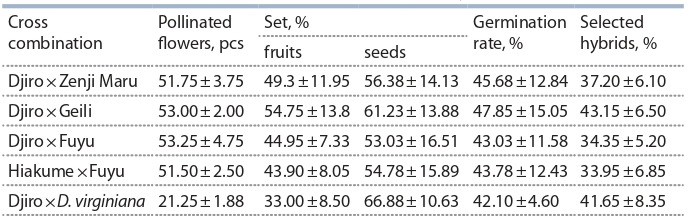
Results of crossing kaki persimmon (average for 4 years)

**Fig. 10.**
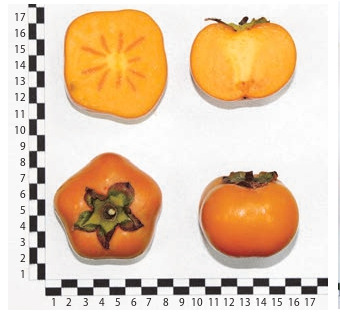
Kaki persimmon cultivar MVG Omarova.

**Fig. 11.**
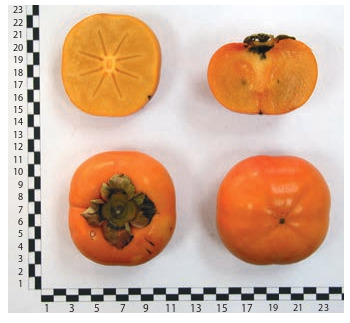
Hybrid form No. 39.

The hybrid fund of kaki persimmon includes 56 forms from intervarietal and interspecific crosses, 18 of which were recorded as promising ones. A new winterhardy cultivar MVG Omarova with good fruit quality (ascorbic acid 20 mg %, the sum of sugars 22 %) was obtained and in 2021 transferred to the “State Register of Selection Achievements Authorized for Use for Production Purposes” of the Russian Federation, (Fig. 10). Currently, the hybrid form No. 39 is being tested by the State Cultivar Commission (Fig. 11).

Feijoa (Feijoa sellowiana Berg) In recent years, in the subtropical regions of Russia, there has been an intensive expansion of feijoa plantings, but mainly by plants grown from seeds without varietal membership. In order to expand the assortment and develop new adapted cultivars, eight cross combinations were carried out, and a large hybrid fund adapted to local cultivation conditions was created (Table 4).

**Table 4.**
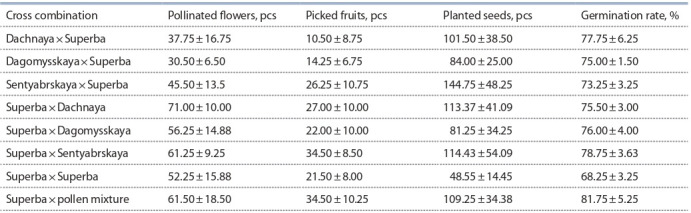
Results of studying feijoa hybrids (average for 4 years)

The best cross combinations, from which seeds with a high germination rate were obtained, are the following: Dachnaya× Superba, Superba×Dagomysskaya and Superba×pollen mixture. From the entire variety of forms, 36 promising ones were recorded, 7 of which are elite with high and stable yields (Table 5).

**Table 5.**
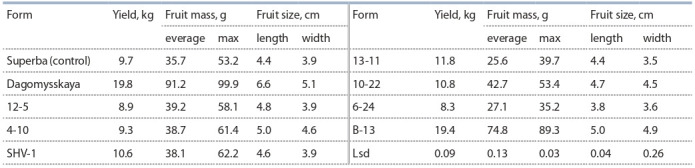
Productivity of feijoa forms aged 10 years

Of these, the form B-13 was recorded as large-fruited, form 10-22 has active growth, and equals the zoned cultivar Superba in fruit mass. 

**Fig. 12.**
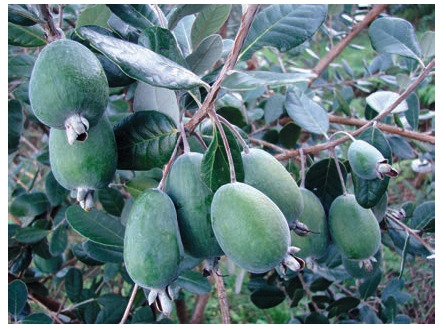
Feijoa cultivar Dachnaya

**Fig. 13.**
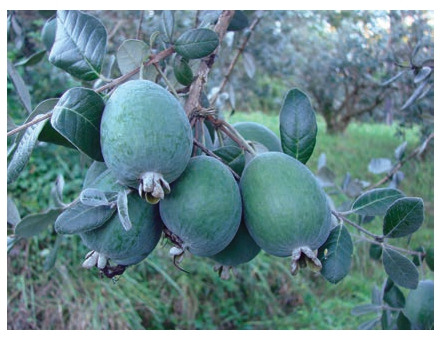
Feijoa cultivar Dagomysskaya

New feijoa cultivars bred by FRC SSC of RAS Dachnaya (Fig. 12), Dagomysskaya (Fig. 13) and Sentyabrskaya are actively used in the breeding process as donors of such signs as “active growth” and “early maturity”.

Tea plant (Camellia sinensis (L.) Kuntze)

**Fig. 14.**
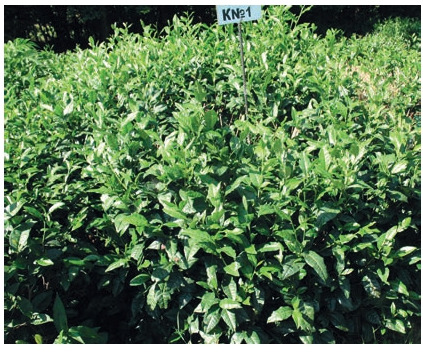
Tea plant, form AF-3

**Fig. 15.**
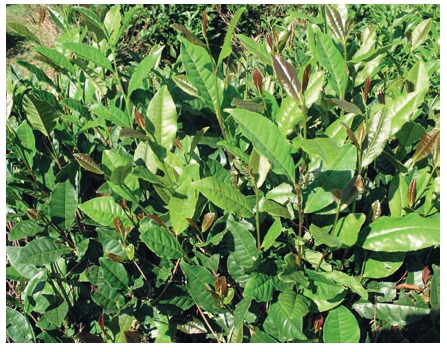
Tea plant, form AF-5

Krasnodar Territory is the northernmost region on the globe where tea culture is cultivated on an industrial scale (Ryndin, Tereshkin, 2012). Research on the development of new cultivars is carried out at FRC SSC of RAS in order to improve winter hardiness, yield and quality of raw materials (Vavilova, 2018). As a result of the work carried out, a promising material with high economic and biological characteristics was recorded. The study includes 38 forms, 3 of which (13-09, 13-13, 13-23) were recorded as candidates for cultivars with high yield (799 g/bush). Five more forms, AF-1, AF-2, AF-3 (Fig. 14), AF-4, AF-5 (Fig. 15), with high winter hardiness, resistance to unfavorable growing conditions and stable yield (423 g/bush) were recorded on the basis of Adygei Branch of FRC SSC of RAS.

As a result of the long-term creation and comprehensive study of the flower crops breeding material, highly decorative and resistant forms were recorded for use in industrial and amateur floriculture, as well as in breeding as sources of valuable traits for creating new cultivars.

The common freesia (Freesia refracta (Jacq.) Klatt) is one of the most popular early spring cultures grown for cut flowers. Hybrid forms of freesia are characterized by a wide range of colours from white, blue, beige, to dark blue, purple, and dark red (Table 6). Spots, smears, strokes, and throat colour located on the surface of the perianth lobes give special originality to the colour shades (Paschenko, 2020a, b). 

**Table 6.**
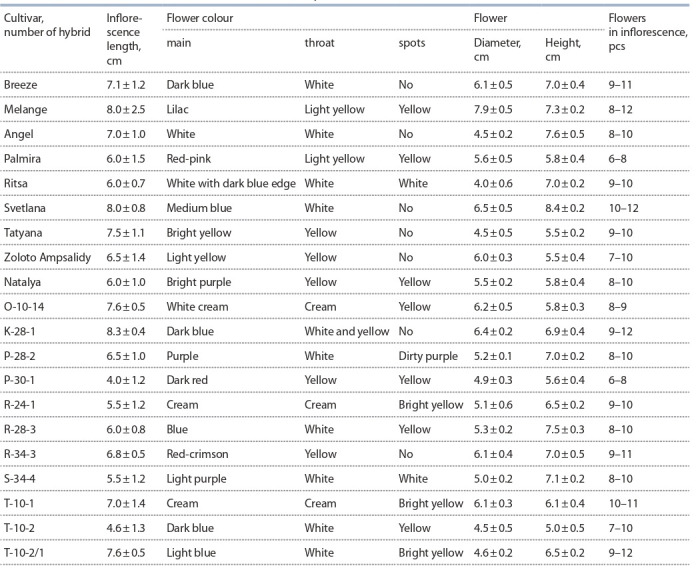
Characteristics of new domestic cultivars and elite hybrid forms of freesia

**Fig. 16.**
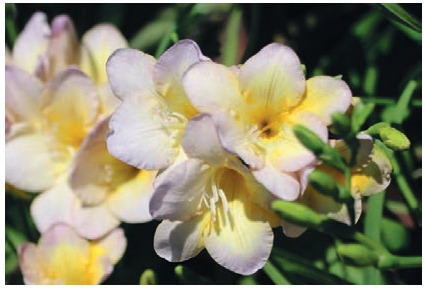
Freesia cultivar Melange.

**Fig. 17.**
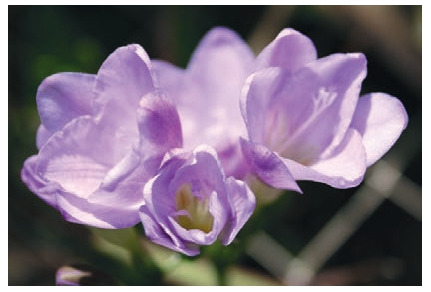
Freesia cultivar Svetlana.

The recorded cultivars and selected elite forms have high decorative qualities of the flower. The colour is varied, ranging from bright white, lilac-yellow, pink-purple to red-crimson and dark blue. In cultivars Breeze, Melange (Fig. 16), Svetlana (Fig. 17) and hybrids K-28-1, R-34-3, T-10-1 and T-10-2/1, the number of flowers in the inflorescence exceeds 10 pcs, the longest inflorescences are in the cultivars Melange and Svetlana, respectively, 8.0±2.5 and 8.0±0.8 cm and in the hybrid form K-28-1 (8.0±0.8 cm).

**Fig. 18.**
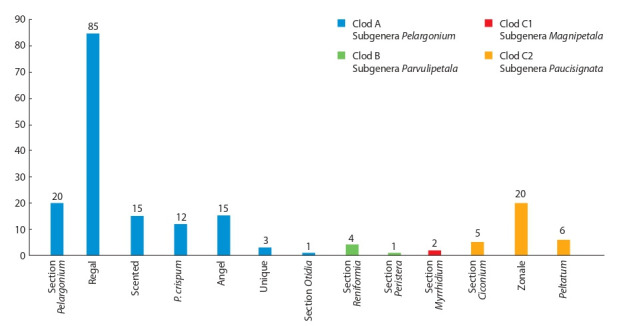
Composition of Pelargonium collection at FRC SSC of RAS.

Pelargoniums (Pelargonium L’Hér. ex Ait.) are the most valuable decorative and deciduous plants. Their significant variety allows to use them for decorating gardens, parks, terraces, balconies, etc. from spring to late autumn. They differ from many ornamental plants in their abundant flowering, resistance to stress factors, and high reproduction rate (Van der Walt, Boucer, 1986; Van der Walt, Vorster, 1988). FRC SSC of RAS has an extensive collection of pelargoniums (200 cultivar samples), which includes representatives of four clods (A, B, C1 and C2), 4 subgenera and 6 sections (Fig. 18). Most of the collection (about 70 %) consists of representatives of the subgenus Pelargonium L’Hér. – these are wild-growing species, including those based on which many modern large-flowered and fragrant pelargonium cultivars, angels and unicums have been obtained (Gutiyeva, 2018).

**Fig. 19.**
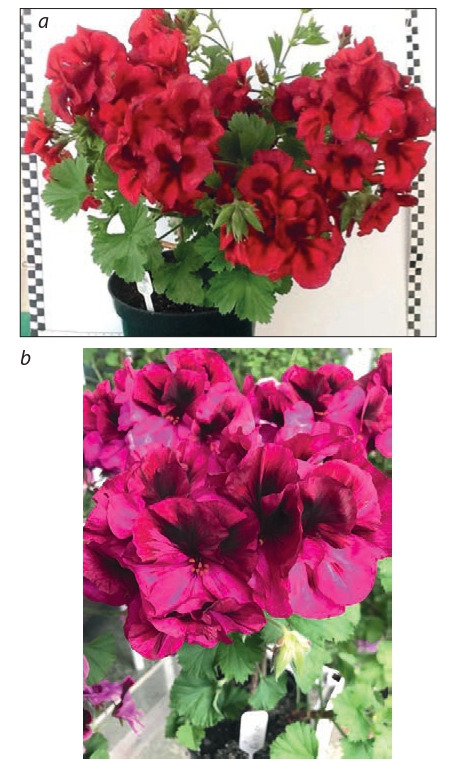
Large flower pelargonium cultivars: a – Carmen suita, b – Flamenko.

**Fig. 20.**
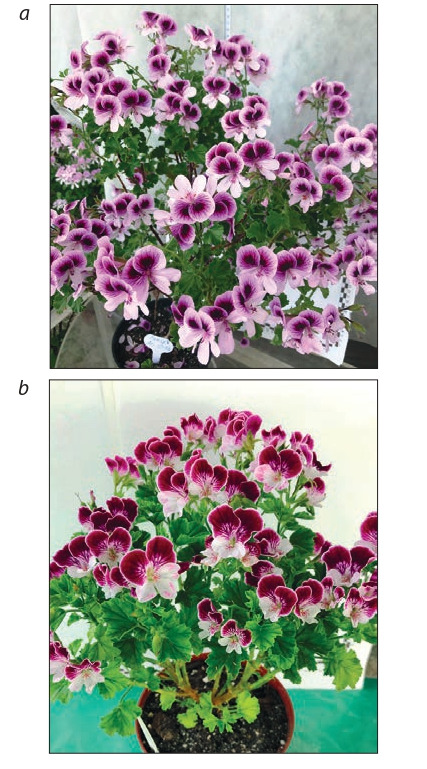
Crisped-leaf pelargonium cultivars: a – Surprise, b – Zagadka.

Federal Research Centre the Subtropical Scientific Centre is carrying out breeding work on these groups using interspecific and intervarietal hybridization and intends to create adaptive, highly decorative, productive, and long-flowering cultivars with various flowering periods for universal use. More than 20 cross combinations were carried out. The nature of inheritance of the main decorative features in flower was determined. It was found that 60 % of the seedlings of the studied cross combinations inherited the maternal colour type. Decorative hybrids, carriers of various useful traits, including a fragrance with a high level of adaptability, were isolated from the hybrid offspring (Table 7, Fig. 19, 20).

**Table 7.**
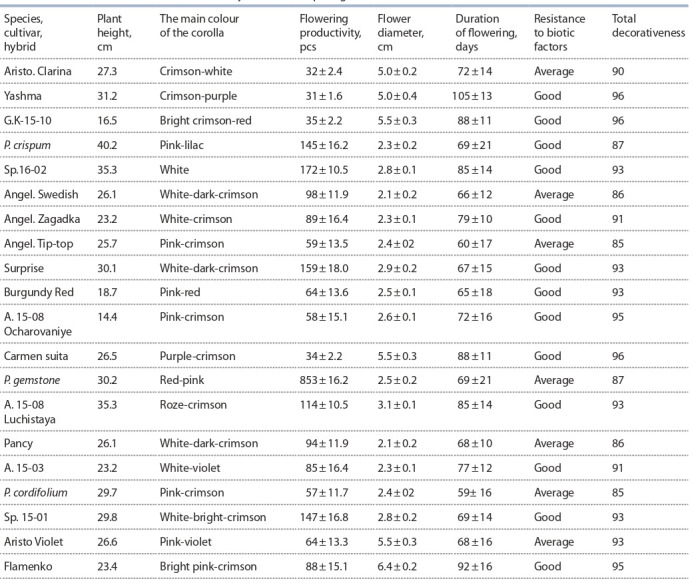
Characteristics of cultivars and elite hybrid forms of pelargonium

Сrown anemone (Anemone coronaria L.) is a perennial herb with an openwork decorative rosette leaves and relatively strong peduncles, 10–40 cm high. Flowers are 6–10 cm in diameter, diverse in shape and colour, with a long (up to 2.5 months) flowering period. Anemone is used in landscaping as a pot culture, in forcing and as a cut flower. 

**Fig. 21.**
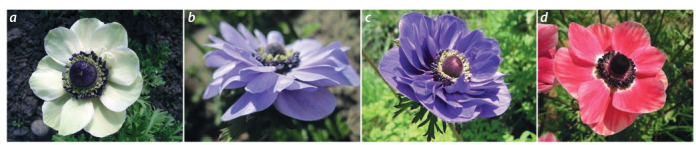
Crown anemone cultivars: a – Polina; b – Flora; c – Volshebstvo; d – Letenitsa

The collection of FRC SSC of RAS includes 25 anemone cultivars, 8 of which are foreign and 17 are domestic. The cultivars selected by the FRC SSC of RAS are distinguished by the diversity and richness in the colour of the perianth lobes, peduncle height and by the productive flowering. The main direction of work with this crop is the creation of cultivars for obtaining cut flower products. The production of new hybrid forms was carried out by intersort hybridization. Criteria for the selection of elite hybrid forms are as follows: a new colour of the corolla or a different combination of colours compared to the original forms; the diameter of the flower more than 6.5 cm; long (more than 25 cm) and stable peduncle; flowering productivity (the number of flowers per plant is more than 8); resistance to abio- and biotic factors (Table 8, Fig. 21).

**Table 8.**
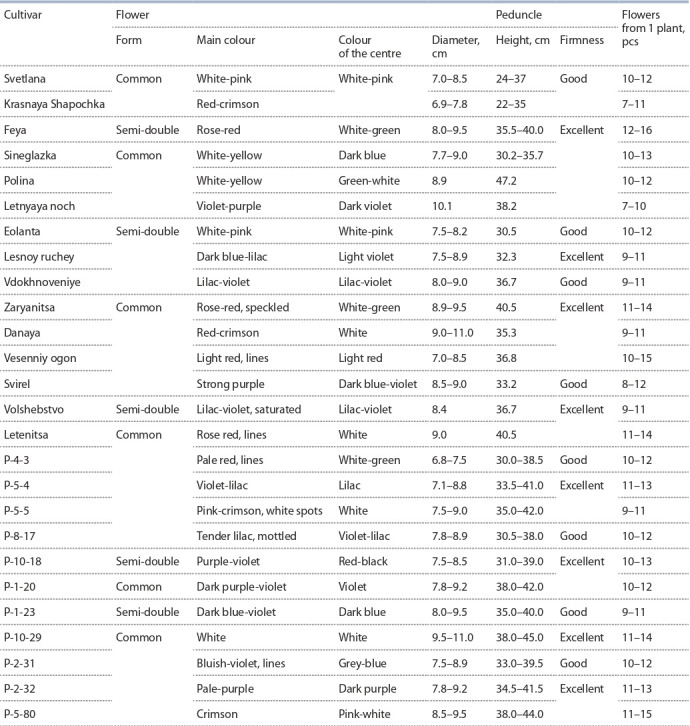
Characteristics of cultivars and hybrid forms of crown anemone

Garden chrysanthemum (Chrysanthemum×hortorum Bailey) is a perennial herbaceous crop that ranks second among cut flowers in terms of economic indicators. Modern cultivars differ in the shape, size and color of the inflorescences, in the height of peduncles, the shape of bush, and in flowering terms. Breeding cultivars that meet international standards and are adapted to the conditions of the humid subtropics in Russia is extremely important. 

The hybrid fund of the FRC SSC of RAS includes 62 chrysanthemum forms, 10 of which are promising and 6 are elite (R-192-4, I-34-5, R-192-12, R-196-4, R-194-13, R-192-12). Two hybrid forms have been prepared for transfer to the State Cultivar Commission of the Russian Federation: R-196-4 and R-192-4. They have a high productivity of 75–125 pcs/m2 and a long flowering period (30–35 days).

## Conclusion

The analysis of results of using various methods in breeding subtropical and flower crops at FRC SSC of RAS showed that the most effective methods are remote and intervarietal hybridization, clonal selection, selection of spontaneous mutations and selection of promising forms from open pollination.

Currently, the FRC SSC of RAS has a rich breeding fund of subtropical, southern fruit and flower plants, from which 989 forms have already been selected for further comprehensive study. Over the past five years, 50 new cultivars have been created and submitted to the State Cultivar Commission, including 26 cultivars of pelargonium, 15 – anemone, 5 – freesia, 2 – chrysanthemum, 1 – kaki persimmon and 1 – apple. In the “State Register of Selection Achievements Authorized for Use for Production Purposes” of the Russian Federation have been included 63 cultivars bred by FRC SSC of RAS, including 26 cultivars of pelargonium, 13 – anemone, 9 – chrysanthemum, 7 – freesia, 4 – hazelnut, 3 – feijoa, 1 – tea plant. 46 patents for breeding achievements were obtained.

Forty-seven sources of economically valuable traits were recorded, including 10 sources for citrus crops, 9 – for pelargonium, 8 – for freesia, 5 – for pear, 4 – for chrysanthemum, 4 – for kaki persimmon, 2 – each for anemone, tulip, kiwi fruit, and 1 – for feijoa.

New cultivars and hybrid forms bred by FRC SSC of RAS show a high adaptation degree to the specific natural and climatic conditions in the region, which distinguishes them from many introduced cultivars and makes it possible to replenish the zoned assortment; furthermore, they present a great interest for further breeding work. Some of them are used in production and landscaping not only in the region, but also in other areas in the south of Russia.

## Conflict of interest

The authors declare no conflict of interest.
